# Current Concepts in the Nonoperative Management of Achilles Tendon Pathologies: A Scoping Review

**DOI:** 10.3390/jcm14134736

**Published:** 2025-07-04

**Authors:** Jennifer A. Kipp, Cody D. Blazek

**Affiliations:** 1 Department of Orthopaedic Surgery and Rehabilitation, Atrium Health Wake Forest Baptist Medical Center, Wake Forest University School of Medicine, Winston-Salem, NC 27101, USA; 2 Department of Vascular and Endovascular Surgery, Atrium Health Wake Forest Baptist Medical Center, Wake Forest University School of Medicine, Winston-Salem, NC 27101, USA

**Keywords:** Achilles tendon, Achilles tendinosis, Achilles rupture, equinus, nonoperative, treatment

## Abstract

**Background/Objectives**: Achilles tendon pathologies, such as Achilles tendinitis, tendinosis, ruptures, and equinus contracture, cause pain and functional impairment. While surgical intervention is indicated in some cases, many patients are successfully managed with nonoperative treatment. The goal of this review was to evaluate the current evidence-based treatments for the nonoperative management of Achilles tendon disorders, focusing on indications and clinical outcomes. **Methods:** A scoping review of the literature was conducted from 2015 to 2025 from the PubMed database. Research published in the last ten years was included if it addressed nonoperative treatments for Achilles tendinopathy, acute ruptures, and/or equinus contracture. The outcome measures of interest included functional outcomes, re-rupture rates, and overall patient satisfaction. **Results:** Nonoperative management results in favorable outcomes for a wide range of Achilles tendon pathologies. Eccentric loading is supported for chronic tendinopathy, and functional rehabilitation programs with early mobilization have shown comparable outcomes to surgical repair for acute tendon ruptures. Combination therapy for the nonoperative management of equinus is favored. These therapies include stretching protocols, casting, and the botulinum toxin. **Conclusions:** The literature supports the notion that nonoperative management strategies for Achilles tendon pathologies provide symptom relief and functional improvement in patients. However, these treatment plans should be individualized and tailored to patient-specific goals.

## 1. Introduction

The Achilles tendon is the largest and strongest tendon in the human body. Formed by the gastrocnemius and soleus muscles, it plays a vital role in foot plantarflexion and ankle stabilization during the gait cycle. Despite its strength, the Achilles tendon is prone to both acute and chronic overuse injuries due to repetitive high mechanical loads. The gastrocnemius muscle has two heads that originate from the posterior femur and spans the knee, ankle, and subtalar joints. The soleus muscle is the primary plantarflexory force of the ankle joint and arises from the posterior tibia and proximal fibula. These muscles function independently based on knee position and unite at the mid-calf to form the Achilles tendon [1]. Together, these two muscles are often referred to as the gastrocnemius soleus complex.

The plantaris muscle is a small, variable muscle with a long tendon that arises on the popliteal surface of the femur. Absent in approximately 10% of individuals, it contributes to the Achilles tendon medially in about 22% of cases [2]. Together, the gastrocnemius soleus complex, as well as the plantaris muscle, make up the superior posterior compartment of the leg. The Achilles tendon becomes a single structure approximately 5–6 cm proximal to its calcaneal insertion and the fibers begin to spiral 12–15 cm above the insertion. The fibers rotate nearly 90°, with the medial fibers orienting posteriorly and the lateral fibers rotating laterally [1].

Achilles tendon disorders are common musculoskeletal conditions that significantly impact mobility and quality of life. A recent study reported an annual incidence of Achilles tendon rupture as high as 40 cases per 100,000 individuals [3]. Achilles tendonitis is an inflammatory condition that is often associated with overuse or a sudden increase in activity [4]. Achilles tendinopathy, or tendinosis, is a degenerative condition that describes pain and sometimes the loss of function with tendon-loading activities. This condition affects both athletic and sedentary populations. The incidence rate is approximately 2–3 per 1000 in the general population, but increases to 5.0–10.9% in runners [5,6]. Equinus is defined as limited ankle dorsiflexion and can affect the biomechanics of the foot and ankle. This condition can be caused by multiple etiologies, including osseous limitations, neuromuscular disorders, or the contracture of the gastrocnemius and soleus muscles and the surrounding soft tissues. Equinus is associated with many lower-extremity pathologies, including plantar fasciitis, Achilles tendonitis, cerebral palsy, strokes, medial tibial stress syndrome, and posterior tibial tendon dysfunction due to altered biomechanics and an increased strain on the posterior chain [7].

The surgical management of these conditions is indicated in some cases, such as cases involving complete tendon rupture or in patients presenting with symptoms that do not respond to conservative management. However, nonoperative treatment is often a first-line approach that can be effective across many presentations. Additionally, there has been a trend toward the nonoperative management of Achilles tendon ruptures [8,9,10]. A scoping review was chosen to summarize the large body of evidence surrounding nonoperative management strategies, given the diversity of conditions and variability in clinical approaches. This format allowed for a broad, yet structured, synthesis across studies for Achilles tendonitis, tendinosis, ruptures, and equinus.

## 2. Materials and Methods

This is a scoping review of the literature on nonoperative treatment for Achilles tendon pathologies and is intended to provide a broad and clinically relevant overview of the available evidence without a formal meta-analysis. This review was created without a pre-registered protocol. A targeted literature search was conducted using the online PubMed database for peer-reviewed studies published from 2015 to 2025. PubMed was selected given its wide coverage in peer-reviewed biomedical and clinical journals. The following search terms were used: “Achilles tendon” OR “Achilles rupture” OR “Achilles tendinopathy” OR “Achilles tendonitis” OR “equinus.” Additionally, “nonoperative” OR “conservative” OR “non-surgical” terms were searched. The literature searches were limited to English-language publications and human studies.

Peer-reviewed studies were included in this review if they were published in the last 10 years, focused on the nonoperative management of Achilles tendonitis, tendinosis, ruptures, or equinus, and had clearly defined methods and outcome measures. Randomized controlled trials, cohort studies, case-controlled studies, and systemic reviews were all included in the literature search. Studies that did not investigate outcomes related to the nonoperative treatment of the beforementioned pathologies, animal studies, and non-peer-reviewed sources were excluded from the analysis. Articles were included based on the lead author’s assessment of their clinical relevance to conservative Achilles tendon pathology management, the transparency of the outcomes, and the overall study quality, as determined through a full-text review.

A data-charting form was developed to systematically gather important information from each included study. The titles and abstracts were initially screened for relevance. Following the initial screening was a full-text review. The articles were categorized into groups based on the condition and its respective treatment. For each study, the author information, publication year, study design, population, sample size, intervention type, outcome measures, and main findings were extracted. No assumptions were made beyond what was reported in the cited articles.

A narrative synthesis was used to organize and interpret the findings from the reviewed literature. The charted data were used to create summary tables and guide the narrative synthesis. The studies were grouped by the Achilles tendon pathology and were examined for trends in the treatments, the outcomes, and their clinical relevance. Similarities and discrepancies between the studies were identified and used to highlight both areas of consensus and gaps in the evidence. The data were compiled into summary tables for comparison. A formal critical appraisal of individual sources was not conducted; however, the study design, sample size, and clinical relevance were all considered when determining which articles to include in the final analysis.

The extracted data were grouped by condition (rupture, tendinopathy, equinus) and intervention type. The key points were summarized and populated into tables to allow for a comparison across the included studies. A meta-analysis was not performed secondary to the heterogeneity in the study designs, populations, and outcome measures.

**Figure 1:**
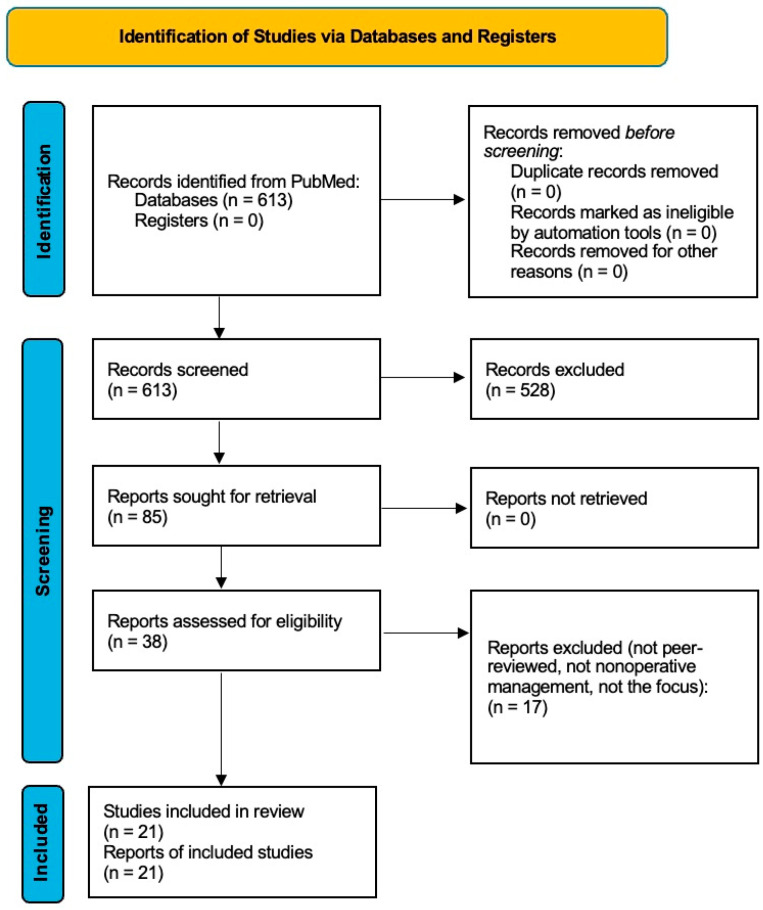
PRISMA scoping review flow diagram of study-selection process.

A total of 613 studies were identified through PubMed. After the screening of the title and abstract, 85 articles remained. Following a full-text review, 21 were included in the final analysis (Figure 1). Sixty-four full-text articles were excluded after reviewing due to a lack of relevance to nonoperative treatment, a focus on surgical management, or insufficient outcome data.

## 3. Results

A total of 613 studies were identified through the PubMed database search. Ultimately, 21 met the inclusion criteria and were included in the primary analysis of this current review. A larger number of studies met the inclusion criteria than were ultimately included in the detailed synthesis. All the studies addressed nonoperative treatments for Achilles tendon rupture, tendinopathy, or equinus. Randomized controlled trials, systematic reviews, and prospective cohort studies were all included. The study selections were based on clinical relevance, the methodological quality, and the focus of this topic. This approach allowed for a focused, yet comprehensive, analysis that aligned with the review’s objectives. The characteristics of the included studies are summarized in Table 1, Table 2, Table 3 and Table 4, including the design, population, and primary outcomes.

### 3.1. Achilles Tendon Rupture

#### 3.1.1. Operative Versus Nonoperative Treatment Outcomes

Several high-quality studies, including randomized controlled trials and systematic reviews, compared the operative to nonoperative management of Achilles tendon ruptures (Table 1). Myhrvoid et al. (2022) reported no significant difference in the functional outcomes between age groups. The nonoperatively treated group did, however, experience a higher re-rupture rate (6.2%) compared to the surgically repaired group (0.6%). Fewer complications were also reported among the nonoperatively treated patients [8]. Similarly, Deng et al. (2017) reported that surgically fixed Achilles tendon ruptures had a statistically significant lower chance of re-rupture than those treated nonoperatively, and that there was no difference in the long-term functional outcomes [9]. A meta-analysis that included over 15,000 patients reported an increase in the tendon re-rupture risk, but a lower complication rate in nonoperatively treated patients. This analysis also supports the integration of a functional rehabilitation program in conservatively treated tendon ruptures [13]. In the athletic population, the findings were similar, and the authors reported a high rate of return to sports. The calf strength was found to be 10–18% higher in the surgically repaired population than the non-surgically treated group at the 18-month follow-up. Younger or athletic individuals may still consider operative repair based on their activity goals and shared decision making [12].

#### 3.1.2. Comparison of Nonoperative Treatment Strategies

When analyzing the outcomes of the methods for the nonoperative treatment of Achilles tendon ruptures, early functional rehabilitation programs resulted in more favorable outcomes than traditional casting methods (Table 2). Two studies published in the last decade evaluated early rehabilitation protocols for acute Achilles tendon ruptures. A multicenter randomized control trial performed by Costa et al. (2020) [14] compared traditional plaster casting to functional bracing in 540 patients who suffered Achilles tendon ruptures. Patients in the functional bracing group were placed into a removable orthosis with heel wedges to allow for plantarflexion. The heel wedges were periodically removed until the patients had progressed to weightbearing in a plantigrade position. At 9 months post-injury, no differences were found in the Achilles tendon total rupture score (ATRS), a measure of physical function. The functional bracing group returned to work sooner and had fewer complications [14].

Ecker et al. [15] conducted a case series that included 114 patients who were prospectively followed for 17 years after nonoperative treatment of Achilles tendon ruptures. The patients were allowed early weightbearing in a walking boot with gradual adjustments in dorsiflexion. This rehabilitation protocol focused on early mobilization while still being mindful of the healing tendon. Over this study’s 17 years of follow-up, the authors reported 11 (9.6%) re-ruptures. Additionally, 90% of the patients reported their outcome satisfaction as “good” or “very good” [15].

Glazebrook et al. described an early functional rehabilitation program for nonoperatively treated Achilles tendon ruptures. The protocol first requires immediate immobilization followed by a gradual progression to weightbearing and controlled exercises. This begins with early diagnosis and careful patient selection. The authors stress that, if a nonoperative treatment functional rehabilitation program cannot be closely monitored and completed correctly, then operative treatment should be considered [29]. While this was not a comparable outcome study, it highlights that early functional rehabilitation offers favorable outcomes in the nonoperative treatment of these ruptures, and that patient selection remains key when choosing between operative versus nonoperative intervention.

### 3.2. Achilles Tendonitis and Tendinopathy

Achilles tendon disorders can be divided into both tendonitis, an acute inflammatory condition, and tendinopathy, chronic tendon degeneration. While tendonitis specifically refers to acute inflammation of the tendon, recent high-powered studies specific to this are scarce, as much of the literature focuses on Achilles tendinopathy. The higher-powered literature evaluates the treatment of the chronic or subacute presentation of the broader category, tendinopathy. Given the limited literature focused solely on tendonitis and the clinical overlap between both tendonitis and tendinosis, the nonoperative treatments for both conditions will be discussed under the umbrella of tendinopathy.

A total of six studies were reviewed to evaluate the nonoperative treatment of insertional and midportion (non-insertional) Achilles tendinopathy. The findings are summarized in Table 3. Prudêncio et al. conducted a systematic review and concluded that eccentric exercise was the most effective treatment for improving pain and function for midportion tendinopathy [16].

Rhim et al. (2020) supports eccentric exercise as well for this condition, but suggests that the addition of a high-volume injection with corticosteroids or extracorporeal shockwave therapy (ESWT) may improve the long-term outcomes in Achilles tendinopathy [17]. Conversely, Kearney et al. (2021) found that platelet-rich plasma (PRP) injection therapy was not found to improve the midportion tendinopathy symptoms when compared to a placebo [18].

In the treatment of insertional Achilles tendinopathy, Mansur et al. (2021) completed a double-blinded randomized control trial and did not determine a difference in the outcomes between a combination of eccentric exercise and ESWT, and eccentric exercise alone [19]. A 2023 systematic review and network meta-analysis found that ESWT and eccentric loading were among the most effective short-term treatments for insertional tendinopathy [20]. Lastly, Zhi et al. (2021) found that ESWT offered the greatest improvement in pain and function, while NSAIDs, physical therapy, heel lifts, and orthoses were less effective adjunctive treatments, but still beneficial [21]. Overall, eccentric loading and ESWT were the most frequently supported modalities for Achilles tendinopathy.

### 3.3. Equinus

After reviewing the recent peer-reviewed literature, the majority of the high-quality studies on the nonoperative treatment of equinus contracture (limited ankle dorsiflexion) were found to mainly focus on neuromuscular populations. Table 4 summarizes the peer-reviewed literature on the nonoperative treatment of equinus deformities in this group [22,23,24,25,26,27,28]. The patient populations, interventions, and key outcomes are highlighted in the table.

Systematic reviews and clinical trials support serial casting, therapy, ankle–foot orthoses (AFOs), and botulinum toxin injections to improve ankle dorsiflexion and gait function. Kumar et al. and Klaewkasikum et al. reported that combination therapies, such as the botulinum toxin with casting or orthoses, can provide superior outcomes compared to monotherapy [22,24]. Chen et al. (2017) found that an adjustable splint-assisted AFO is effective at improving the passive range of motion and gait function in patients with cerebral palsy [23]. ESWT was found to reduce spasticity and improve ankle mobility in patients following a stroke [26]. Tustin and Patel (2017) determined that serial casting provides short-term improvements in mobility, but noted that adjunctive treatments are needed for the long-term maintenance of a correction [25]. Campinini et al. (2022) reports gait improvement in stroke patients with the use of AFO braces and stretching exercises [27].

Muzaffar et al. (2017) [28] evaluated a device called a tilt board with target stretching (TBTS), which is designed to allow for the self-stretching of the gastrocnemius and soleus muscles. Their results showed significant improvement in ankle equinus with regular use. The authors of this prospective randomized control trial concluded that this home-based stretching tool is effective for the conservative management of equinus contractures [28].

Table 5 provides an overview of the reviewed treatments for Achilles tendon pathologies. This includes the primary conservative treatment recommendations, which are based on the current review of the literature. Additionally, key considerations are mentioned.

## 4. Discussion

The nonoperative management of Achilles tendon ruptures have become increasingly validated in the recent literature, especially in conjunction with a rehabilitation plan. Historically, nonoperative treatment was not favored due to concern about unfavorable outcomes. However, recent high-powered studies have challenged this notion, citing that operatively and nonoperatively treated Achilles ruptures may have comparable outcomes in many patients. As with any treatment, it is important to stress that the decision for operative versus nonoperative management is patient-specific in every situation.

A 2022 multicenter randomized controlled trial found no differences in the functional outcomes between the operative and nonoperative management of Achilles ruptures. They did report a higher re-rupture rate in the nonoperative group (6.2% versus 0.6%) [8]. Ochen et al. cited a higher re-rupture rate in nonoperatively treated ruptures as well, but also highlighted the fewer complications in the nonoperative management cohort [13]. These findings highlight the support for nonoperative management coupled with a rehabilitation program as a reasonable treatment for Achilles tendon ruptures, especially in patients who are less active or have comorbidities that increase their surgical risk. If electing for nonoperative management, early mobilization has been found to be advantageous over traditional casting and immobilization [14,29].

An important consideration in reviewing this literature is the variability in the characteristics of the ruptures and treatment regimens across studies. Not all of the studies specified the rupture location or severity. There is literature supporting that the rupture gap size does not influence the nonoperatively treated outcomes [30]. However, Yassin et al. (2020) cited poorer patient-reported outcomes for nonoperatively treated tendon ruptures withgaps greater than 5 mm in the athletic population and greater than 10 mm in the non-athletic population [31]. The time to weightbearing after injury also differed in these studies. Additionally, patient selection remains an important factor. Many studies excluded high-level athletes, and as a result, their findings may not be generalizable to patient populations with high demands. Data for longer follow-up periods are available in some studies, but are still relatively limited and may not fully capture tendon durability in the long term. Glazebrook et al. (2019) stressed the importance of adherence to a functional rehabilitation program [15]. If patients are unable to adhere to said program, surgical repair may be a more reliable option.

Achilles tendinopathy and tendonitis are prevalent conditions that cause functional limitations. Eccentric loading exercises remain the most consistently supported intervention for tendinopathy, especially midportion. Eccentric exercises can decrease local pain by strengthening the gastrocnemius and soleus muscles and lengthening the myotendinous unit [21].

Adjunctive therapies, such as ESWT, have also shown promising results for tendinopathy treatment. The proposed mechanisms of the positive effects observed for ESWT use in tendinopathy include the facilitation of collagen synthesis as well as the upregulation of growth factors that promote tenocyte proliferation. Multiple factors are attributed to the analgesic effect, including pain neurotransmission and nociceptor hyperstimulation [32]. PRP injections have gained popularity as a potential tendinopathy treatment, due to their growth factors that may accelerate healing. Similar to ESWT, the mechanism behind PRP is not fully understood, but the platelets are thought to stimulate healing by releasing signaling proteins at the site of the injury [33]. Some retrospective reviews suggest an advantage of using PRP. However, studies with a higher level of evidence do not support a significant efficacy for this treatment [34,35,36].

Rhim et al. support eccentric exercise for midportion Achilles tendinopathy and suggest that adjunctive treatments, such as corticosteroid injections or ESWT, may improve the long-term outcomes [17]. The 2024 Academy of Orthopaedic Physical Therapy (AOPT)/Journal of Orthopaedic Sports Physical Therapy (JOSPT) clinical practice guidelines for Achilles tendinopathy also recommend eccentric exercise as a first-line treatment. Activities should be modified to patient tolerance, but complete immobilization is discouraged. They recommend stretching for patients with limited dorsiflexion, but advise against night splints, heel lifts, or passive modalities as standalone treatments. The guidelines do not recommend corticosteroid injections due to potential adverse effects on the tendon structure, including the risk of rupture. Shockwave therapy is considered a viable adjunctive therapy to eccentric loading programs [37]. A randomized controlled trial by Kearney et al. (2021) found that platelet-rich plasma (PRP) injection therapy did not provide a significant benefit in Achilles tendinopathy treatment over a placebo, consistent with the AOPT/JOSPT guideline’s position that PRP should not be a first-line treatment due to inconsistent evidence [18,37].

Achilles tendonitis, an acute inflammatory condition, is often cited clinically, but there are limited studies that have investigated this phase of Achilles tendon pathology in isolation. Many studies have grouped this into the “tendinopathy” category. As a result, this review evaluated evidence that addresses tendinopathy as a whole with the understanding that activity modification, early intervention, and rehabilitation are effective across both pathologies. Additionally, many of the cited studies support the use of a multimodal approach in treatment protocols.

This review only included literature published within the last ten years. High-quality, recent evidence related to the nonoperative treatment of equinus in the general population is scarce. As a result, the studies in this review focused on the treatment of patients with neuromuscular conditions. Systematic reviews and clinical trials report that interventions such as botulinum toxin injections, serial casting, ESWT, and AFOs are all effective at improving ankle dorsiflexion.

Given that the recent literature for conservative equinus treatment is largely based on the neurologic population, older studies remain relevant in current clinical practice. Grady and Saxena (1991) published one of the earlier clinical studies that support a dedicated gastrocnemius stretching program for the improvement of ankle dorsiflexion [38]. Macklin et al. demonstrated substantial improvements in ankle range of motion and gait following an eight-week stretching program in runners with equinus [39]. Radford et al. (2006) confirmed that static stretching increases dorsiflexion across various patient populations [40]. These findings support the biomechanical rationale behind stretching as a treatment for equinus, particularly for non-neurologic presentations.

Both recent and earlier studies underscore the value of nonoperative treatment for equinus contracture. While much of the literature focuses on neurologic populations, available evidence, particularly regarding stretching protocols, also support its use in patients without neuromuscular conditions. 

An important consideration when evaluating nonoperative treatment strategies for Achilles tendon pathologies is the rate and rationale for surgical intervention. A subset of patients may ultimately require surgery due to persistent symptoms or initial treatment failure, and conversion rates vary by condition and patient population. For example, Achilles tendon ruptures may require surgical intervention in the setting of a re-rupture, or in patients that have an inability to adhere to a prescribed nonoperative rehabilitation program [8]. The nonoperative management of Achilles ruptures is effective in the general population, but younger or athletic individuals that have higher physical demands may opt for a surgical intervention to reduce their re-rupture risk [10,11]. For Achilles tendinopathy, approximately 80% of patients experience a resolution of their symptoms without surgery [41]. Compliance also plays an important role in the success of conservative treatment. Those who fail to adhere to functional rehabilitation protocols are more likely to experience suboptimal outcomes, which ultimately may progress to surgery [15].

Differences in patient outcomes are observed across specific populations. For example, elderly patients tend to tolerate nonoperative protocols well and may benefit from the lower complication rates associated with the conservative management of Achilles tendon pathologies. This is especially true in the setting of comorbidities [9]. Conversely, individuals with higher functional demands may have better subjective and functional outcomes with the operative management of acute Achilles tendon ruptures [10]. Patients with diabetes face increased risks in terms of delayed healing, postoperative infections, and impaired collagen remodeling [42,43]. These factors stress the importance of an individualized treatment plan that accounts for comorbidities and patient goals.

This narrative review is subject to several limitations. First, a qualitative synthesis is provided, rather than a systematic or meta-analytic evaluation for the treatment of Achilles tendon pathologies. The study selection may have been influenced by author discretion and may not have encompassed all the relevant literature. The lead author selected the studies based on their relevance to nonoperative Achilles tendon pathologies, the clarity of the outcomes, and the methodological quality, as determined through a full-text review. The absence of multiple independent reviewers may have introduced subjectivity into the selection process. Additionally, the selection of studies was limited to peer-reviewed publications from the last ten years, which may have excluded earlier high-quality studies still relevant to clinical care. In an attempt to address this gap, older literature was discussed outside of the primary analysis. Finally, the included studies ranged in terms of their patient populations, causes of pathology, interventions, and outcomes measures. As a result, this can limit the direct comparison of the results, and subsequently may reduce the generalizability of the treatments across the many included subgroup populations. Future research may help clarify the treatment protocols and improve the consistency of outcome reporting for nonoperative management strategies.

## 5. Conclusions

Achilles tendon pathologies encompass a wide range of conditions, from acute injury to chronic degeneration. The evidence supports early functional rehabilitation for acute ruptures and eccentric loading for tendinopathy. Surgical intervention remains an appropriate option in some cases, particularly for complete ruptures as well as conditions that do not improve with conservative treatment. The treatment should reflect the specific condition and patient profile and recognize that there is a role for both conservative care and surgical intervention.

## Figures and Tables

**Table 1:** Operative versus nonoperative treatment.

Citation	Study Design	Sample Size	Treatment	Primary Outcomes	Key Findings
Myhrvold et al., [8]	Multicenter RCT	554	Nonoperative vs. surgical treatment	ATRS, re-rupture rate, complications	No difference in functional outcomes; higher re-rupture rate in non-op group (6.2%) vs. surgical group (0.6%); fewer complications in non-op group.
Deng et al. [9]	Systematic review and meta-analysis	Pooled data	Surgical vs. conservative	Re-rupture rate, complications, return to activity	Surgery had lower re-rupture rate, but more complications; no difference in long-term function.
Lantto et al. [10]	Prospective RCT	60	Surgical vs. nonoperative with rehab	ATRS, re-rupture, strength	Similar function; slightly better strength in surgery group; fewer complications in non-op group.
Seow et al. [11]	Systematic review and meta-analyses	Meta-analysis of meta-analyses	Surgical vs. conservative	Re-rupture, complications, function	Surgery had lower re-rupture rate, but higher complications. Functional rehab was important in non-op group for successful outcomes.
Lerch et al. [12]	Retrospective cohort	89	Nonoperative with early rehab	Return to sport, satisfaction	70% returned to sport; 67% for high-activity patients. High patient satisfaction overall.
Ochen et al. [13]	Systematic review and meta-analysis	15,862	Surgical vs. nonoperative (with and without rehab)	Re-rupture, complications	Surgery had significantly lower re-rupture rate (2.3% vs. 3.9%), but more complications. In patients with early rehab, re-rupture rates were similar.

Abbreviation: ATRS, Achilles tendon rupture score.

**Table 2:** Achilles rupture nonoperative treatment protocols.

Citation	Study Design	Sample Size	Intervention	Weightbearing	Primary Outcomes	Key Findings
Costa et al. (UK STAR Trial) [14]	Multicenter randomized control trial	540	Functional bracing vs. plaster casting	Full weightbearing allowed within 48 h with brace	ATRS, return to work, complications	Higher ATRS, earlier return to work, and fewer complications in bracing group
Ecker et al. [15]	Prospective case series	114	Early weightbearing protocol using walking boot	Immediate weightbearing with progressive dorsiflexion	Functional outcomes, re-rupture rates	Low re-rupture rate and favorable functional recovery with early mobilization

Abbreviation: ATRS, Achilles tendon total rupture score.

**Table 3:** Achilles tendinosis treatment.

Citation	Study Design	Condition Focus	Intervention	Key Findings
Prudêncio et al. [16]	Systematic review and meta-analysis	Midportion tendinopathy	Various conservative treatments	Eccentric exercise was most effective at improving pain and function.
Rhim et al. [17]	Network meta-analysis	Midportion tendinopathy	HVI, ESWT, eccentric loading	Adding HVI with corticosteroids or ESWT to eccentric exercise may improve long-term outcomes.
Kearney et al. [18]	Randomized clinical trial	Midportion tendinopathy	PRP injection vs. placebo injection	No difference in outcomes between both groups.
Mansur et al. [19]	Double-blinded randomized clinical trial	Insertional tendinopathy	ESWT and eccentric exercise vs. eccentric exercise alone	No difference in outcomes between both groups.
Ko et al. [20]	Systematic review and network meta-analysis	Insertional tendinopathy	Eccentric exercise, ESWT, cryotherapy, orthotics	ESWT and eccentric loading were among the most effective short-term treatments.
Zhi et al. [21]	Systematic review	Insertional tendinopathy	NSAIDs, physical therapy, ESWT, heel lifts, orthoses	ESWT was most effective; combining with physical therapy was also beneficial.

Abbreviations: HVI, high-volume injection; ESWT, extracorporeal shockwave therapy; PRP, platelet-rich plasma.

**Table 4:** Equinus nonoperative treatment.

Citation	Study Design	Population	Intervention	Key Findings
Kumar et al. [22]	Systematic review and meta-analysis	Children with spastic cerebral palsy	Botulinum toxin + casting	Improved ankle dorsiflexion and gait short-term; combination therapy was more effective than either alone.
Chen et al. [23]	Prospective cohort	Children with cerebral palsy	Adjustable splint-assisted AFO	Orthotic use improved passive ROM and gait parameters.
Klaewkasikum et al. [24]	Systematic review and meta-analysis	Children with spastic CP and equinus gait	Various conservative treatments	Conservative approaches (casting, AFO, stretching) significantly improved dorsiflexion and gait.
Tustin & Patel [25]	Narrative review	Children with cerebral palsy	Serial casting	Temporary gains in dorsiflexion; adjunct therapies needed for sustained results.
Yang et al. [26]	Randomized controlled trial	Stroke patients with plantar flexor spasticity	ESWT	ESWT reduced spasticity and increased ankle dorsiflexion.
Campanini et al. [27]	Scoping review	Stroke patients with equinus	Physical therapy modalities (e.g., stretching, AFO)	PT strategies were generally effective at reducing triceps spasticity and improving gait.
Muzzafar et al. [28]	Randomized controlled trial	Patients with equinus, both spastic and non-spastic	TBTS control	Significant decrease in equinus after 1 month of TBTS use.

Abbreviations: AFO, ankle–foot orthosis; CP, cerebral palsy; ESWT, extracorporeal shockwave therapy; TBTS, tilt board with target stretching; ROM, range of motion.

**Table 5:** Achilles tendon pathology treatments summarized.

Achilles Tendon Pathology	Treatment	Key Considerations
Tendon Rupture: Operative Fixation	Follow surgeon’s postoperative rehabilitation protocol.	Lower re-rupture rate.
Tendon Rupture: Conservative Treatment	Early functional rehabilitation. If concerned about compliance with rehab protocol, consider operative fixation.	Higher re-rupture rate; fewer complications.
Achilles Tendinosis	Eccentric exercise as primary treatment; ESWT or PRP injections may be considered as secondary modalities.	Eccentric exercise is most consistently supported as the superior treatment option.
Equinus	Serial casting, static stretching, and dynamic bracing.	Treatment is patient-specific and guided by the underlying cause of contracture. Long-term effectiveness may vary.

Abbreviations: ESWT, extracorporeal shockwave therapy; PRP, platelet-rich plasma.

## Data Availability

This article is a comprehensive review and does not include any original data. The sources of all the derived data are cited throughout the manuscript.

## Abbreviations

The following abbreviations are used in this manuscript:

ESWT	Extracorporeal shockwave therapy
NSAID	Nonsteroidal anti-inflammatory drug
AFO	Ankle–foot orthoses
TBTS	Tilt board with target stretching
PRP	Platelet-rich plasma
